# A nonparametric spatial scan statistic for continuous data

**DOI:** 10.1186/s12942-015-0024-6

**Published:** 2015-10-20

**Authors:** Inkyung Jung, Ho Jin Cho

**Affiliations:** Department of Biostatistics and Medical Informatics, Yonsei University College of Medicine, 50-1 Yonsei-ro, Seodaemun-gu, Seoul, 120-752 Korea

**Keywords:** Spatial cluster detection test, Normal-based scan statistic, Wilcoxon rank-sum test

## Abstract

**Background:**

Spatial scan statistics are widely used for spatial cluster detection, and several parametric models exist. For continuous data, a normal-based scan statistic can be used. However, the performance of the model has not been fully evaluated for non-normal data.

**Methods:**

We propose a nonparametric spatial scan statistic based on the Wilcoxon rank-sum test statistic and compared the performance of the method with parametric models via a simulation study under various scenarios.

**Results:**

The nonparametric method outperforms the normal-based scan statistic in terms of power and accuracy in almost all cases under consideration in the simulation study.

**Conclusion:**

The proposed nonparametric spatial scan statistic is therefore an excellent alternative to the normal model for continuous data and is especially useful for data following skewed or heavy-tailed distributions.

## Background

Geographic disease surveillance involves identifying areas with unusually high or low rates of disease outcome. One of the most widely used methods is the spatial scan statistic, which has been developed for several different probability models such as Poisson, Bernoulli, ordinal, multinomial, exponential, and normal. The most popular disease outcome is of count data type such as disease incidence or mortality, which can be analyzed using the Poisson model when the number of cases is compared to underlying population [1]. For case–control type of data, the Bernoulli model is used [1]. Multi-category disease outcomes such as disease subtypes or cancer stage can be analyzed using multinomial or ordinal models [2–4]. The exponential model is used for spatial cluster detection for survival time data [5]. For continuous data such as birth weight in infants, a spatial scan statistic based on the normal probability model has been proposed [6]. For continuous regional measures at geographic levels such as mortality rate at the county level, a weighted normal model, which considers the weights reflecting the uncertainty of the regional measures or sample size, has been proposed [7].

Here we focus on continuous outcome data and propose a nonparametric spatial scan statistic that does not require distributional assumption. The normal and weighted normal models are parametric methods based on the normal distribution. These models can be used for non-normal data because they still maintain the correct significance by using the permutation procedure for obtaining a p-value as indicated by Kulldorff et al. [6]. However, the statistical power of the models has not been fully evaluated for non-normal data. In this paper, we develop a nonparametric spatial scan statistic based on the Wilcoxon rank-sum test statistic and compare the performance of the method with parametric models via a simulation study under various scenarios.

While the parametric spatial scan statistic is the maximum value of the likelihood ratio test statistics comparing inside versus outside a scanning window over numerous windows, the proposed nonparametric spatial scan statistic is defined as the minimum of p-values from Wilcoxon rank-sum tests. We also use the permutation procedure for evaluating the statistical significance of the detected cluster. Therefore, the proposed method also maintains the significance level correctly. Through a thorough simulation study we evaluate the performance of the method. The proposed method is compared to the normal-based spatial scan statistic in terms of conventional statistical power, indicated as conditional power by Takahashi and Tango [8], and accuracy measures of sensitivity and positive predicted value (PPV). Extended power proposed by Takahashi and Tango [8] is also presented.

## Methods

### A scan statistic for continuous data based on the normal probability model

Suppose that we have continuous outcome data such as birth weight at each location of a study region and that we want to identify an area with a higher (or lower) mean of outcome than remaining areas. Kulldorff et al. [6] proposed a scan statistic for continuous data based on the normal probability model for this kind of problem. The null hypothesis of no clustering is written as $H_{0} :\mu = \eta$ for all *z* and the alternative is $H_{a} :\mu > \eta \ (\textrm{or } \mu < \eta )$ for some *z*, where *μ* and *η* are the means of outcome variables inside and outside scanning window *z*, respectively. A large number of scanning windows with variable sizes are imposed on a study region and each scanning window is a candidate for the most likely cluster. Circular scanning windows are considered here.

The normal distribution has two parameters of mean and variance. Kulldorff et al. [6] assumed a common variance inside and outside the scanning window under the alternative hypothesis. Given window *z*, the log-likelihood ratio test statistic *LLR*(*z*), equivalent to the likelihood ratio test statistic *LR*(*z*), is given by

$$\begin{aligned} LLR\left( z \right) = \log LR\left( z \right) = {\text{N}}ln\left( {{\hat{{\sigma }}}} \right) + \mathop \sum \limits_{i} \frac{{\left( {x_{i} - \hat{\mu }} \right)^{2} }}{{2{\hat{{\sigma }}}^{2} }}  - \frac{N}{2} - Nln\left( {\sqrt {{\hat{{\sigma }}}_{z}^{2} } } \right) \end{aligned}$$

where *N* is the total number of observations, *x*_*i*_ are the continuous observations (*i* = 1,…, *N*), $\hat{\mu } = \sum\nolimits_{i} {x_{i} } /N$ and ${\hat{{\sigma }}}^{2} = \sum\nolimits_{i} {\left( {x_{i} - \hat{\mu }} \right)^{2} /N}$ are the maximum likelihood estimates (MLEs) of the mean and variance under the null hypothesis, respectively, and ${\hat{{\sigma }}}_{z}^{2}$ is the MLE of the common variance under the alternative hypothesis, which is given by

$${\hat{{\sigma }}}_{z}^{2} = \frac{1}{N}\left\{ {\mathop \sum \limits_{i \in z} \left( {x_{i} - \hat{\mu }_{z} } \right)^{2} + \mathop \sum \limits_{i \notin z} \left( {x_{i} - \hat{\eta }_{z} } \right)^{2} } \right\}.$$

Here, $\hat{\mu }_{z} = \sum\nolimits_{i \in z} {x_{i} /n_{z} }$ and $\hat{\eta }_{z} = \sum\nolimits_{i \notin z} {x_{i} /\left( {N - n_{z} } \right)}$ are the MLEs of the mean parameters under the alternative hypothesis, where $n_{z}$ is the number of observations inside window *z*. The *LLR*(*z*) depends on *z* only through the last term, and therefore, the most likely cluster is the area that minimizes the variance under the alternative hypothesis, which in turn maximizes *LLR*(*z*).

To evaluate the statistical significance of the most likely cluster, randomly permuted data sets are generated and the maximum of *LLR*(*z*) is calculated for each data set. The p-value of the most likely cluster is computed as the rank of the maximum of *LLR*(*z*) from the original data set among all data sets divided by the number of all data sets. All procedures for finding the most likely cluster and obtaining the p-value have been implemented into the SaTScan software [9].

Kulldorff et al. [6] applied the normal-based scan method to New York City birth weight data, and identified two statistically significant clusters of low birth weight that corresponded to areas with high infant mortality. These authors suggested that the normal model could be used for a wide variety of continuous data, which may not be normally distributed, however it was not recommended for exponential or other types of survival data. Kulldorff et al. [6] further mentioned that the correct type I error rate will be maintained even for non-normal data due to the permutation procedure. However, the statistical power of detecting clusters for non-normal data has not been evaluated.

### A nonparametric spatial scan statistic

Here we proposed a nonparametric spatial scan statistic for continuous outcome data, which requires no distributional assumptions. The null hypothesis is written as $H_{0} :F_{in} = F_{out}$ for all *z* and the alternative is $H_{a} :F_{in} \left( x \right) = F_{out} \left( {x - \Delta } \right)$ for some *z*, where $F_{in}$ and $F_{out}$ are the cumulative distribution functions (cdfs) of outcome variable inside and outside scanning window *z* and $\Delta$ is a location shift of the cdf for outside relative to inside *z*. A positive $\Delta$ implies that outcomes tend to be higher inside compared to outside *z* and a negative $\Delta$ indicates the inverse outcome. We propose to use the Wilcoxon rank-sum test statistic as the test statistic for the nonparametric spatial scan statistic. Specifically, we compute the Wilcoxon rank-sum test statistic for a given scanning window comparing inside versus outside *z* and obtain a p-value, and the minimum of p-values over all scanning windows is the test statistic. The area associated with the smallest p-value is defined as the most likely cluster. Calculation of the Wilcoxon rank-sum test and a p-value is quite simple. Assign ranks to the observations, using the average rank in the case of tied observations, and suppose that the rank of *x*_*i*_ is *R*_*i*_ (*i* = 1, …, *N*). The Wilcoxon rank-sum test given *z* is $W_{z} = \sum\nolimits_{i \in z} {R_{i} }$ and a p-value can be obtained using the normal approximation for $W_{z}$. Under $H_{0}$, $E(W_{z} ) = n_{z} \left( {N + 1} \right)/2$ and $Var(W_{z} ) = n_{z} \left( {N - n_{z} } \right)\left( {N + 1} \right)/12$. For $n_{z} \ge 10$ and $\left( {{\text{N}} - n_{z} } \right) \ge 10$, $T_{z} = \left( {W - E(W_{z} )} \right)/\sqrt {Var(W_{z} )}$ is approximately normally distributed with a mean of 0 and a variance of 1 [10]. Therefore, the test statistic given *z* is $1 - {{\varPhi }}(T_{z} )$ for $\Delta > 0$ and ${{\varPhi }}(T_{z} )$ for $\Delta < 0$, where ${{\varPhi }}$ is the cdf of the standard normal distribution. For small values of $n_{z}$ or ${\text{N}} - n_{z}$, the exact method to compute a p-value can be used [10].

We also use the same permutation procedure as the normal-based scan statistic to evaluate the statistical significance of the most likely cluster. In addition to the most likely cluster, we also report secondary clusters with statistical significance, if any, when they have no geographical overlap with more significant clusters.

### Simulation study settings and performance measures

To evaluate the statistical power and accuracy of the proposed nonparametric spatial scan statistic and the normal-based method, we conducted a simulation study under various scenarios. Assuming several different distributions, we created a true cluster that tends to have higher outcomes than the remaining areas on an 8 × 8 unitless grid with a length of two units for each side of a cell. The center of the true cluster was at the coordinates 11 and 5, and any cell whose center is within the radius of a length of 3 was included in the true cluster. In this way, the true cluster consisted of 9 cells, the center cell at the 6th column from left and the 3rd row from the bottom and 8 cells around the center cell. We considered normal, logistic, double-exponential, uniform, lognormal, t-, and Cauchy distributions. Under each distribution, location parameters were set different inside and outside the true cluster. For normal, logistic, double-exponential, and uniform distributions, the mean of the distributions was set to $c \sqrt 2$ (*c* = 0.5, 1, 1.5) inside and 0 outside the cluster. For lognormal distributions, the mean was set to 2 +  $c \sqrt 2$ (*c *= 0.5, 1, 1.5) inside and 2 outside the cluster since the mean of lognormal distributions cannot be zero. The variance was set to 1 over all areas for normal, logistic, double-exponential, uniform, and log normal distributions. For t-distributions, the degrees of freedom was set to 3 and the mean difference between inside and outside the cluster was $c \sqrt 2$ (*c* = 0.5, 1, 1.5). For Cauchy distributions, we set the scale parameter to 1 and the location parameter to *c* = 2, 4, 6, inside and 0 outside the cluster, because the Cauchy distribution does not have mean and variance.

We generated 1000 data sets of sample size 64 for each scenario and tested whether there was a cluster that tended to have higher outcomes than remaining areas for each of the 1000 simulated data sets, using the proposed method and the normal-based method. The statistical power was estimated as the number of rejected data sets out of 1000 at the significance level of 0.05. We also estimated sensitivity and PPV in order to evaluate the accuracy of the detected cluster. Sensitivity was defined as the proportion of the number of cells correctly detected among the cells in the true cluster and PPV as the proportion of the number of cells belonging to the true cluster among the cells in the detected cluster. Sensitivity and PPV were estimated as the average of the proportions only for data sets rejected at the significance level of 0.05.

Although power, sensitivity, and PPV are well-defined and useful measures for comparing the performance of spatial scan statistics [2–6], another useful tool is the extended power and its profile proposed by Takahashi and Tango [8]. The extended power is defined as a weighted sum of a bivariate power function, P(1,s) of “length” *l*, which is the size of the detected cluster (i.e. the number of cells in the detected cluster), and “include” *s*, which is the number of cells belonging to the true cluster among the cells in the detected cluster. In our simulation setting, P(9,9) indicates the power of exactly detecting the true cluster. The weight function includes penalties for false positives (FPs) and false negatives (FNs) and the extended power is expressed as a function of the penalties. Using certain penalties, the extended power is reduced to the usual power. Takahashi and Tango [8] proposed to use the profile of the extended power which represents the extended power continuously for all values of the ratio of penalties, *r*. Further details on the extended power can be found in the paper by Takahashi and Tango [8], and an example of its application was reported by Guttmann et al. [11]. We also presented profiles of the extended power using the results from the simulation studies.

## Results

**Fig. 1:**
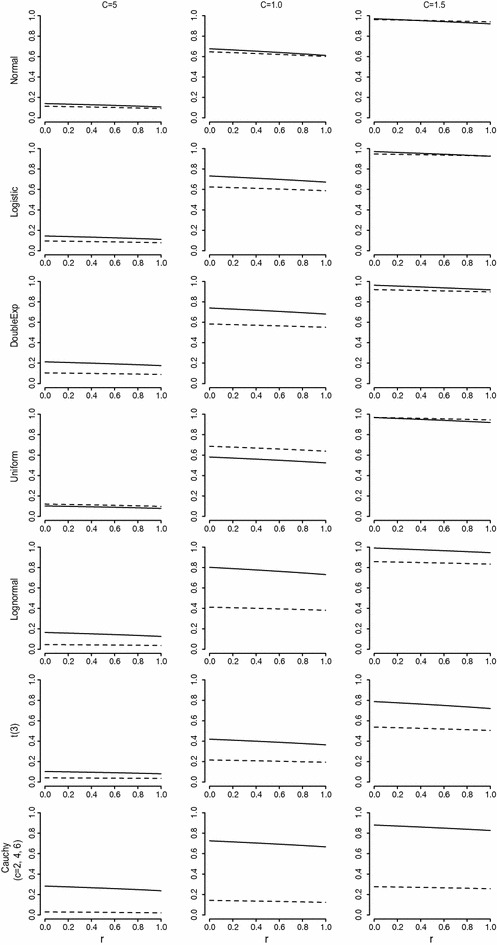
Profiles of the extended power for the nonparametric (*solid line*) and the normal-based (*dashed line*) spatial scan statistic under various distributions

Table 1 shows the estimated power, sensitivity, and PPV for the proposed method and the normal-based method. In most cases, except for uniform distributions, the power of the nonparametric method was higher than that of the normal-based method. Even for normal distributions, the nonparametric method demonstrated slightly higher power than the normal-based method. Although the power of both methods becomes higher as the difference in location parameters inside versus outside the cluster gets larger, the power of the nonparametric method was much higher, especially for very heavy-tailed distributions such as t(3) and Cauchy, as well as for asymmetric distributions such as lognormal. Sensitivity was also higher for the nonparametric method than the normal-based method in most cases. In cases of uniform distribution, the sensitivity of the nonparametric method was as good as the normal-based method even though the power was slightly lower. The PPV of the nonparametric method was similar to or slightly lower than the normal-based method in most cases. This may be because clusters detected using the normal-based method are rather smaller than clusters identified using the nonparametric method. Sensitivity and PPV depend on the size of detected clusters. Therefore, the profile of the extended power presented in Fig. 1 can be very useful for describing overall performance. We used the quantities for the values associated with penalties for FN and FP as proposed by Tango and Takahashi [8]. As shown in Fig. 1, the nonparametric method is uniformly more powerful than the normal-based method in every case except for uniform distributions. The extended power of the nonparametric method was much higher than that of the normal-based method, especially for lognormal, t(3), and Cauchy distributions.

**Table 1:** Statistical power, sensitivity, and positive predictive value (PPV) of the nonparametric spatial scan statistic and normal-based method for various distributions

	Power (%)		Sensitivity		PPV	
	Nonparametric	Normal	Nonparametric	Normal	Nonparametric	Normal
*c* = 0.5						
Normal	17.3	14.8	0.71	0.65	0.63	0.65
Logistic	17.7	12.9	0.72	0.64	0.64	0.65
DoubleExp	24.0	13.5	0.81	0.67	0.74	0.72
Uniform	13.4	15.4	0.65	0.66	0.62	0.69
Lognormal	19.7	7.6	0.74	0.50	0.64	0.52
t(3)	13.9	7.6	0.66	0.44	0.59	0.55
Cauchy (*c* = 2)	31.4	5.7	0.83	0.44	0.76	0.38
*c* = 1.0						
Normal	71.8	69.8	0.90	0.87	0.85	0.89
Logistic	76.9	66.7	0.91	0.89	0.88	0.91
DoubleExp	76.9	62.1	0.93	0.89	0.88	0.91
Uniform	62.2	74.8	0.88	0.86	0.85	0.89
Lognormal	83.2	45.0	0.93	0.86	0.87	0.87
t(3)	45.8	25.9	0.86	0.75	0.80	0.80
Cauchy (*c* = 4)	76.1	16.9	0.92	0.79	0.88	0.74
*c* = 1.5						
Normal	98.6	98.4	0.97	0.96	0.92	0.96
Logistic	98.8	96.8	0.97	0.96	0.93	0.97
DoubleExp	97.6	94.1	0.97	0.96	0.93	0.96
Uniform	98.4	99.1	0.97	0.96	0.93	0.96
Lognormal	99.8	87.9	0.99	0.96	0.93	0.95
t(3)	83.8	58.8	0.92	0.86	0.87	0.89
Cauchy (*c* = 6)	90.9	30.4	0.94	0.87	0.91	0.85

## Conclusions and discussions

We have proposed a nonparametric spatial scan statistic for continuous data. As seen in simulation studies, the nonparametric model has higher power and precision than the normal model especially for heavy-tailed or asymmetric distributions. It is somewhat surprising that the power is higher even for normal distributions although the difference is not very large. This could be due to the current simulating setting of a relatively small number of data points. Another simulation study involving more varied situations would help to better evaluate the performance of the proposed method; however, we believe that the nonparametric model works very well and can serve as an excellent alternative to the normal model for spatial cluster detection for continuous data.

The proposed method can be applied to a wide range of continuous data such as birth weight, body mass index, and cholesterol level of individuals, except for survival time data, for which methods that can handle censored observations [5] are more suitable. Ordinal type of data with many categories can also be analyzed by the proposed method.

As the parametric normal-based spatial scan statistic, the proposed test statistic was constructed under the assumption of independent observations. However, this does not mean that the test assumes that there is no spatial auto-correlation. As described in SaTScan User Guide [12], it is a test of whether there is spatial auto-correlation or other divergences from the null hypothesis. Spatial auto-correlation should not be adjusted away when we are interested in detecting clusters due to such correlation.

The nonparametric spatial scan statistic can be easily extended to space–time settings by considering a three-dimensional cylindrical scanning window with a base representing space and a height representing time. Considering different shapes for scanning windows other than circles, such as ellipses [13] or irregular shapes [14–16] would also be interesting for the nonparametric spatial scan statistic.
